# Losartan enhances the suppressive effect of pirfenidone on the bleomycin-induced epithelial-mesenchymal transition and oxidative stress in A549 cell line

**DOI:** 10.22038/IJBMS.2023.68982.15035

**Published:** 2023

**Authors:** Arian Amirkhosravi, Mahmoud Reza Heidari, Somayyeh Karami-Mohajeri, Maryam Torshabi, Ali Mandegary, Mehrnaz Mehrabani

**Affiliations:** 1 Physiology Research Center, Institute of Neuropharmacology, Kerman University of Medical Sciences, Kerman, Iran; 2 Department of Toxicology and Pharmacology, Faculty of Pharmacy, Kerman University of Medical Sciences, Kerman, Iran; 3 Pharmaceutics Research Center, Institute of Neuropharmacology, Kerman University of Medical Sciences, Kerman, Iran; 4 Department of Dental Biomaterials, School of Dentistry, Shahid Beheshti University of Medical Sciences, Tehran, Iran; 5 Department of Toxicology and Pharmacology, Faculty of Pharmacy, Shahid Sadoughi University of Medical Sciences, Yazd, Iran

**Keywords:** Bleomycin, Epithelial-mesenchymal – transition, Idiopathic pulmonary – fibrosis, Losartan, Oxidative stress, Pirfenidone

## Abstract

**Objective(s):**

Idiopathic pulmonary fibrosis (IPF) is a fatal lung disease. Despite the promising anti-fibrotic effect, the toleration of pirfenidone (PFD) by the patients in full dose is low. Combination therapy is a method for enhancing the therapeutic efficiency of PFD and decreasing its dose. Therefore, the present study evaluated the effect of a combination of losartan (LOS) and PFD on oxidative stress parameters and the epithelial-mesenchymal transition (EMT) process induced by bleomycin (BLM) in human lung adenocarcinoma A549 cells.

**Materials and Methods:**

The non-toxic concentrations of BLM, LOS, and PFD were assessed by the MTT assay. Malondialdehyde (MDA) and anti-oxidant enzyme activity including catalase (CAT) and superoxide dismutase (SOD) were assessed after co-treatment. Migration and western blot assays were used to evaluate EMT in BLM-exposed A549 after single or combined treatments.

**Results:**

The combination treatment exhibited a remarkable decrease in cellular migration compared with both single and BLM-exposed groups. Furthermore, the combination treatment significantly improved cellular anti-oxidant markers compared with the BLM-treated group. Moreover, combined therapy markedly increased epithelial markers while decreasing mesenchymal markers.

**Conclusion:**

This *in vitro *study revealed that the combination of PFD with LOS might be more protective in pulmonary fibrosis (PF) than single therapy because of its greater efficacy in regulating the EMT process and oxidative stress. The current results might offer a promising therapeutic strategy for the future clinical therapy of lung fibrosis.

## Introduction

Idiopathic pulmonary fibrosis (IPF) is an irreversible, chronic, and progressive age-based disease with unknown causes worldwide. The overall prevalence of IPF is estimated to be 3 to 9 cases per 100,000 person-years in Europe and North America (1). Clinical manifestations include progressive dyspnea combined with dry cough and shortness of breath, leading to respiratory failure and death. IPF has a poor prognosis, with a 3–4 year median survival (2). There is no clear information on the pathophysiological mechanisms underlying IPF. However, it may be caused by repeated micro-injuries of the alveolar epithelium and overproduction of reactive oxygen species (ROS) (3). Resultant ROS can activate transforming growth factor-β (TGF-β), triggering epithelial-mesenchymal transition (EMT) as the main pathological process in IPF (4). The important features of IPF are loss of polarity, cell-cell contact, and remodeling of epithelial cells of the cytoskeleton. There are some key molecular events behind the EMT process including down-regulation of epithelial-type proteins (E-cadherin and Zonula occludens-1 (ZO-1) intermediate filament proteins) and up-regulation of mesenchymal markers (Vimentin, α-smooth muscle actin (α-SMA), etc.). The consequences are inducing cellular migration and accumulation of the extracellular matrix (ECM) in the lung tissue (5). Hence, reducing oxidative stress and blocking the EMT process might be an effective therapeutic approach for attenuation of pulmonary fibrosis (PF). 

Up to now, the anti-fibrotic drug 5-methyl-1-phenyl-2-(1H)-pyridone or pirfenidone (PFD) has been proven by the US Food and Drug Administration (FDA) to treat IPF patients (6). More importantly, this drug is widely used to treat the current global SARS-CoV-2 pandemic (7). According to *in vivo* and *in vitro *studies, PFD with anti-proliferative, anti-oxidant, and anti-fibrotic features postpones IPF via attenuating EMT, collagen deposition, and fibroblast proliferation (8, 9). However, despite the promising anti-fibrotic effect of pirfenidone, its toleration by patients in full doses is low (10). Hence, ongoing efforts have been exploring a new therapeutic strategy or alternative therapy for IPF. 

Combination treatment is one of these choices to overcome the progression of PF regarding its higher therapeutic effects besides lower side effects and drug resistance. Compounds with the same target protein or the same pathway can benefit from combination therapy (11). 

Losartan (LOS) is an angiotensin II receptor antagonist and an effective agent for stabilizing lung function in IPF patients, with a relatively low toxicity profile (12). TGF-β can be increased by angiotensin II during IPF, hence it is fair to believe that blocking the angiotensin II receptor can slow the course of IPF (13). TGF-β binds to TGF-β type I and type II subtypes and forms a complex, which leads to the activation of Smad2 and Smad3. After phosphorylation, Smad2 and Smad3 are combined with Smad 4, then the Smad2/3-Smad4 complex translocates to the nucleus to regulate gene expression by interacting with DNA binding transcription factors. The TGF-β/Smad signaling pathway plays an important role in IPF through the regulation of three processes: Myofibroblast differentiation, EMT, and fibrogenesis **(**14**)**. LOS can hinder the TGF-β/Smad signaling pathway significantly in several different tissues (15, 16). In addition to its anti-fibrotic activity, its low toxicity and adverse effects have made LOS an interesting agent for combination therapy in IPF. 

In the present study, bleomycin (BLM) was used to imitate an *in vitro *model of lung fibrosis. BLM has been widely used in chemotherapy for many years (17). However, its application is restricted due to numerous side effects such as skin and lung fibrosis caused by deficiency of BLM hydrolase enzyme activity in these tissues (18). The BLM has been used in many studies conducted on the cellular and molecular mechanisms involved in the pathogenesis of IPF and the identification of novel therapies for this disease (19). Therefore, the current study aimed to investigate the effect of combining PFD with LOS on the EMT process in an *in vitro *model of lung fibrosis induced by BLM in the A549 cell line.

## Materials and Methods

***Materials***

A549 (adenocarcinoma human alveolar basal epithelial cells, C137, NCBI) was provided by Pasture Institute Cell Bank, Tehran, Iran. Dimethyl sulfoxide (DMSO), thiazolyl blue tetrazolium bromide (MTT), Malondialdehyde (MDA), Phosphate buffered saline (PBS, powder, pH 7.4), and penicillin-streptomycin (P/S) were purchased from Sigma-Aldrich Co. Ltd., St. Louis, MO, USA. Dulbecco’s Modified Eagle’s Medium F12 (DMEM/F12) and fetal bovine serum (FBS) were supplied from Gibco-BRL Co. Ltd., Waltham, MA, USA. Polyvinylidene difluoride membrane (PVDF) was acquired from Bio-Rad Laboratories, CA, USA. EMT antibodies against α-SMA, β- catenin, Vimentin, Zo-1, Snail1, Slug, and E-cadherin were purchased from Cell Signaling Technology Inc., USA. Antibody against GAPDH (Glyceraldehyde 3-phosphate dehydrogenase) was purchased from Santa Cruz Biotechnology, USA. BLM was purchased (MW=1512.6 g/mol) from Selleck Chemicals, Houston, TX, USA, and PFD (MW=185.22 g/mol) from Intermune Company, USA. LOS (MW=461 g/mol) is manufactured by Sigma -Aldrich Co. Ltd., St. Louis, MO, USA.

***Cell culture***

A549 cells were cultured and passaged in DMEM F12 supplemented with 10% inactivated FBS and 1% solution of P/S at 37 °C in a humid atmosphere containing 5% CO_2_. 

*Cell viability assay*

Cell viability was examined by the MTT assay. Briefly, cells were seeded in 96-well plates at 10^4^ cells/100 μl for 24 hr. Next, the cells were treated with various concentrations of BLM (0.05 to 0.5 µg/ml), PFD (0.025 to 0.5 mM), and LOS (0.05 to 1 mM) alone for 48 hr to determine the non-toxic concentrations of compounds. After adding 10 μl of MTT (5 mg/ml) to the wells, it was incubated further three hours at 37 °C and 5% CO_2_. After that, formazan crystals were dissolved in DMSO. The absorbance of each group was recorded (*n*=3) at 570 nm utilizing a microplate reader (ELX800, Bio-Tek, USA). This absorbance is proportional directly to the number of living cells. Finally, results were presented as a percentage of control values. We selected the non-toxic concentration of BLM for further evaluation (0.1 µg/ml). Besides, we chose the non-toxic and tolerated concentration of LOS and PFD on A549 cells for the next steps. Generally, the cells were then divided randomly into five main classes: the control group (treated with medium alone); the BLM-treated group; the BLM-LOS-treated group; the BLM-PFD treated group, and BLM with the combination of LOS and PFD (LOS+PFD)-treated group.

*Scratch assay*

A549 cells were seeded onto a 24-well plate at a density of 6× 10^4^ cells/well to measure cell migration. In this study, using *in vitro *scratch assay, the protective effects of LOS (0.2 and 0.5 mM) and PFD (0.2 mM) on the A549 cell-treated BLM for 48 hr were evaluated and compared. For this purpose, by using a sterile 10-100 µl pipette tip, the cell monolayer culture (~ 100% confluence) was scratched. Then, the cells were rinsed with PBS twice and substituted with fresh medium, either supplemented with test reagents or medium alone (control). After 0 and 48 hr, an inverted microscope was utilized to capture images from five independent, random microscopic fields (20). Image J analysis software was used to measure gap areas and calculate the relative cell migration of the treated to untreated control cells.

***Measurement of MDA content, superoxide dismutase (SOD), and catalase (CAT) activities***

The cells were cultured at 6× 10^4^ in a 24-well plate, and after treatment with BLM and LOS and/or PFD for 48 hr, they were collected to measure oxidative stress markers. MDA concentration was measured by washing the cells with ice-cold PBS and homogenizing them in 400 µl of 0.5% Triton X-100. The cell homogenates were utilized in the TBARS assay. MDA level was measured colorimetrically as the degraded product of thiobarbituric acid at 532 nm (21). Data were expressed as nmol/mg protein.

 For catalase (CAT) determination, the homogenized cells were incubated with H_2_O_2, _and after 10 min, the reaction was stopped by adding ammonium molybdate. H_2_O_2 _is converted by the CAT enzyme into oxygen and water in the samples. The ammonium molybdate forms a yellow complex with the remaining H2O2, detected spectrophotometrically at 410 nm. CAT activity was expressed as nmol/min/mg protein (22).

 To determine the Copper-zinc superoxide dismutase (Cu, Zn-SOD), the ability of the enzyme to hinder the pyrogallol autoxidation was measured. This technique is based on the dismutation of this radical by SOD and the competition between the pyrogallol autoxidation by O_2_•¯. A unit of Cu, Zn-SOD activity is determined as the quantity of enzyme needed for causing 50% inhibition of pyrogallol autoxidation. At 412 nm, absorption was read for SOD in U/ml (23, 24).

***Western blotting***

A549 cells were treated and then homogenized using RIPA (Radioimmunoprecipitation assay) lysis buffer containing phosphatase and protease inhibitor cocktails. The Bradford method was used to measure total protein (25). The cell lysates were combined with an equivalent volume of 2X Laemmli sample buffer. Equal amounts of protein were exposed to sodium dodecyl sulfate-polyacrylamide gel electrophoresis (SDS-PAGE) for separation and transferred onto a 0.2 μm PVDF membrane. It was followed by blocking with skim milk (5%) at room temperature (RT) for 2 hr, incubation of the membranes was performed with the indicated rabbit monoclonal antibodies (1:1000 dilution of primary antibodies incubated at 4 °C overnight, 1:2000 dilution of horseradish peroxidase-conjugated goat anti-rabbit IgG antibodies incubation at RT for 1 hr). ECL (enhanced luminol-based chemiluminescent) western blot detection kit was used to detect protein bands. GAPDH was used as the loading control. All immunoblots were repeated three times independently, and ImageJ software was used to quantify the protein expression levels.

***Statistical analysis***

One-way analysis of variance (ANOVA) was used to assess the statistical analyses after Tukey’s multiple comparisons, then Dunnett’s test for selected pairs if appropriate with Prism 8.0 (GraphPad Software, La Jolla, CA, USA). Data were presented as the mean ± standard deviation (SD). 

## Results

***Effect of BLM, PFD, and LOS on A549 viability***

**Figure 1:**
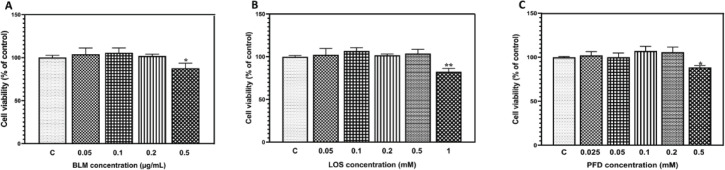
Effect of bleomycin (BLM), pirfenidone (PFD), and losartan (LOS) on A549 viability. The effect of different concentrations of (A) BLM (0.05–0.5 µg/ml), (B) LOS (0.05–1 mM), and (C) PFD (0.025–0.5 mM) on % of cell growth of A549 cell line after 48 hr post-treatment. Results are presented as the mean ± SD (n = 3). *P<0.05 and **P<0.01 significant difference vs the control group

Several concentrations of BLM (0.05, 0.1, 0.2, and 0.5 µg/ml) were used in A549 cells and cell viability was evaluated by MTT assay to determine the optimal concentration of BLM for treating A549 cells to prevent excessive cell death. BLM was non-toxic at a concentration below 0.2 µg/ml, so BLM at 0.1 and 0.2 µg/ml was chosen for the next steps. Furthermore, MTT was used to examine the effect of PFD and LOS on the viability of A549 cells. We measured the cytotoxicity of five serial dilutions of PFD (0.025, 0.05, 0.1, 0.2, and 0.5 mM) and LOS (0.05, 0.1, 0.2, 0.5, and 1 mM) to determine the non-toxic concentrations. It was inferred from the current results that the maximum non-toxic concentration of PFD was 0.2 mM. To introduce LOS as an intervention in BLM-induced EMT, we also selected two non-toxic concentrations (0.2 and 0.5 mM) of LOS alone, as shown in Figure 1. 

***Effect of BLM, PFD, LOS, and PFD+LOS on A549 cells migration***

**Figure 2:**
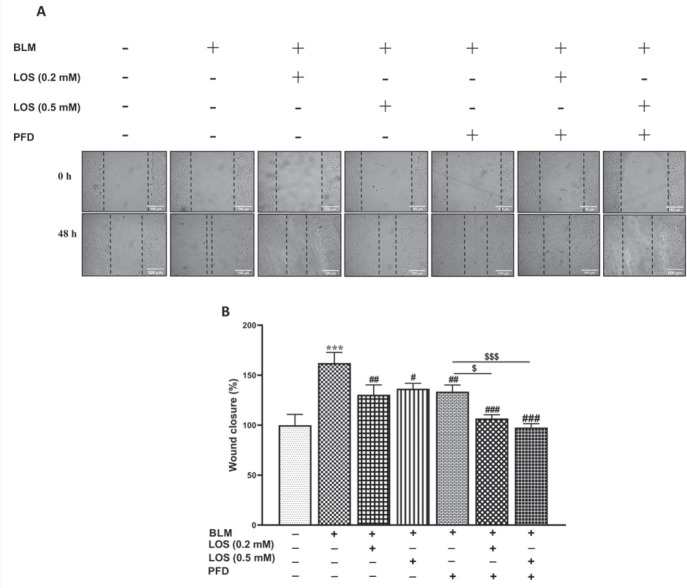
Effect of BLM, PFD, LOS, and combination of pirfenidone with losartan (PFD+LOS) on A549 cells migration by scratch assay. Cells were exposed to BLM (0.1 μg/ml) and co-treated with LOS (0.2–0.5 mM) and PFD (0.2 mM), either alone or in combination. (A) Microscopic images of the scratch assay were captured at 0 (scratch creation) and 48 hr post-scratching/treatment. Graph (B) shows a quantitative analysis of relative cell migration according to the distance between the scratch edges. Cell migration was defined as 100% when cells were cultured with DMEM alone (non-treated control). The migratory ability of A549 cells was decreased significantly by LOS, PFD, and a drug combination consisting of LOS (0.2 mM)+PFD and LOS (0.5 mM)+PFD. Data were expressed as the mean ± SD for at least three independent tests. One-way ANOVA was used for the comparisons between groups. ***P<0.001 significant difference vs the control group; #*P*<0.05, ##*P*<0.01, and ###*P*<0.001 vs the BLM group; &*P*<0.05 and &&&*P*<0.001 vs the PFD group

Among 0.1 and 0.2 μg/ml BLM concentrations, the concentration of 0.1 μg/ml was better through scratch assay (data not shown). As shown in Figure 2, the migratory rate of A549 cells significantly increased by BLM at a concentration of 0.1 μg/ml (162.2 ± 10.6%) compared with the control. However, the migration rate of the BLM group was significantly decreased after exposure to LOS (0.2 mM) (130.4 ± 9.9%), LOS (0.5 mM) (136.5 ± 5.3%), PFD (0.2 mM) (133.5% ± 6.6%), LOS (0.2 mM)+PFD (106.7 ± 3.6%), and LOS (0.5 mM)+PFD (97.6 ± 3.8%,). The decreases in migration were even more noticeable with LOS+PFD. The migration rate of A549 cells exposed to LOS (0.2 mM)+PFD and LOS (0.5 mM)+PFD was lower than those exposed to PFD alone. 

***Effect of BLM, PFD, LOS, and PFD+LOS on A549 oxidative stress parameters***

**Figure 3:**
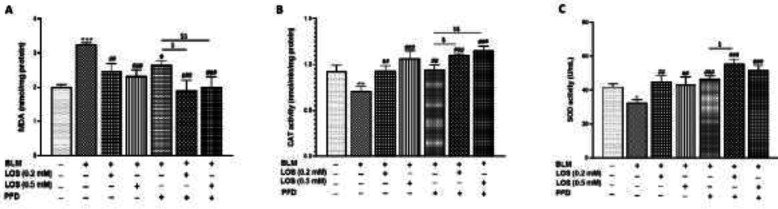
Effect of BLM, PFD, LOS, and combination of pirfenidone with losartan (PFD+LOS) on A549 oxidative stress parameters. Cells were treated with BLM (0.1 μg/ml) in combination with LOS (0.2–0.5 mM), PFD (0.2 mM), or both for 48 hr. The (A) MDA level was decreased significantly by LOS, PFD, and drug combination consisting of LOS (0.2 mM)+PFD and LOS (0.5 mM)+PFD, while (B) CAT and (C) SOD activities were increased. Data are presented as the mean ± SD from at least 3 separate experiments. Comparisons between groups were achieved using ANOVA tests. *P<0.05, **P<0.01, and ***P<0.001 indicate significant differences as compared with the control group; #*P*<0.05, ##*P*<0.01, and ###*P*<0.001 vs the BLM group; $*P*<0.05 and &&*P*<0.01 vs the PFD group

MDA: malondialdehyde; SOD: superoxide dismutase; CAT: catalase; BLM: bleomycin; LOS: losartan; PFD: pirfenidone; Control group: medium alone

After exposure of A549 cells to BLM (0.1 μg/ml), the level of MDA was significantly higher than in the control group. However, treatments significantly reversed the effect of BLM on MDA levels in A549 cells (Figure 3A). BLM also decreased the activity of CAT and SOD enzymes versus the control group. Treatment with LOS (0.2–0.5 mM) and PFD, either alone or with BLM increased CAT and SOD activity (Figure 3, B and C). It seems that the combination of drugs was more effective than the single ones in regulating the oxidative parameters in A549 cells.

***Effect of BLM, PFD, LOS, and PFD+LOS on EMT- involved proteins in A549 cells***

**Figure 4:**
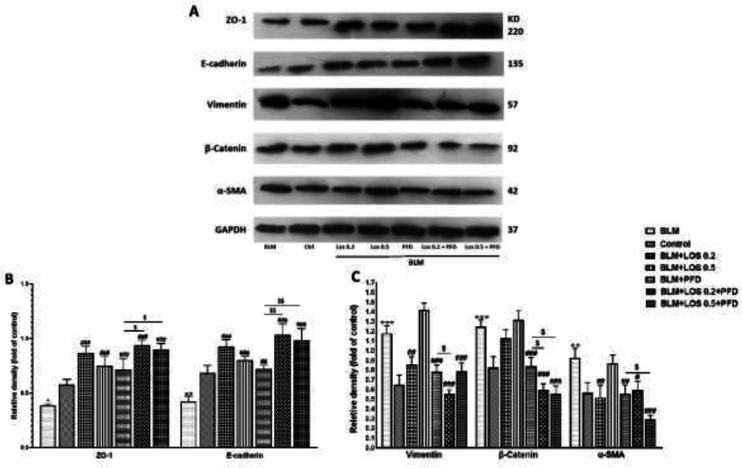
Effect of BLM, PFD, LOS, and combination of pirfenidone with losartan (LOS+PFD) on the expression of ZO-1, E-cadherin, Vimentin, β-Catenin, and α-SMA proteins in A549 Cells. The cells were incubated with LOS (0.2–0.5 mM) and PFD (0.2 mM) while adding the two agents under stimulation with BLM (0.1 μg/ml) induction for 48 hr. The lysates of treated cells were centrifuged and assessed by western blot analysis. The internal reference was GAPDH. (A) Densitometric analysis of the protein expression of ZO-1, E-cadherin, Vimentin, β-Catenin, and α-SMA is presented. ZO-1 and E-cadherin expression were significantly decreased, while Vimentin, β-Catenin, and α-SMA expressions were significantly increased in BLM-stimulated EMT. However, expressions of the epithelial markers were reversed, and the mesenchymal markers were decreased by LOS and PFD treatment, either alone or in combination. (B, C) Quantitative results are presented as mean ± SD of the index's relative protein levels from three independent experiments.*P<0.05 and **P<0.01 significant difference vs the control group; #*P*<0.05, ##*P*<0.01, and ###*P*<0.001 vs the BLM group. $*P*<0.05, and $$*P*<0.01 vs the PFD group

EMT: epithelial-mesenchymal transition; BLM: bleomycin; LOS: losartan; PFD: pirfenidone

According to Figure 4, the epithelial markers’ protein levels such as ZO-1 and E-cadherin were decreased, and the mesenchymal markers including Vimentin, β-Catenin, and α-SMA were dramatically up-regulated in A549 cells after treatment with BLM in comparison with the control. In contrast, LOS and PFD treatment, either alone or in combination, significantly increased the expression of mentioned epithelial markers or decreased the mesenchymal markers in A549 cells compared with the BLM group. 

**Figure 5:**
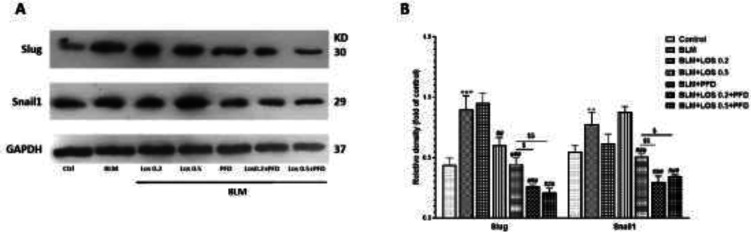
Effect of BLM, PFD, LOS, and combination of pirfenidone with losartan (LOS+PFD) on Slug and Snail1 protein expressions in A549 Cells. The cells were incubated with LOS (0.2-0.5 mM) and PFD (0.2 mM), and the combination of LOS with PFD under stimulation of BLM for 48 hr. The whole cell lysates were immunoblotted with antibodies against Slug and Snail1. GAPDH was used as the internal reference. (A) Representative blots are shown. Slug and Snail1 protein expressions in A549 cells were significantly increased in BLM-induced EMT. LOS, PFD, and drug combination significantly attenuate BLM-mediated EMT. (B) Western blot signal intensities were quantified by ImageJ software. All data represent the mean ± SD from triplicate samples for each treatment. **P<0.01 and ***P<0.001 significant difference vs the control group; ##*P*<0.01 and ###*P*<0.001 vs the BLM group. $*P*<0.05 and $$*P*<0.01 vs the PFD group

EMT: epithelial-mesenchymal transition; BLM: bleomycin; LOS: losartan; PFD: pirfenidone

In Figure 5, we also evaluated the effect of all treatments in the presence of BLM on the expression of EMT-related transcription factors such as Slug and Snail1 in A549 cells. Western blot analyses revealed that Slug and Snail1 expression levels increased significantly in the A549 cells after exposure to BLM (*vs* control). LOS, PFD, and LOS+PFD reduced the expression and activity of above mentioned transcription factors (*vs* BLM group). Together, these results suggest that co-treatment of LOS with PFD had the most pronounced effect on BLM-induced EMT in A549 cells.

## Discussion

IPF is a complicated and lethal lung disease that occurs due to collagen decomposition in alveolar cell walls (26). PFD was approved for treating IPF; however, it has lower toleration by the patients in full dose (10). Therefore, there is an emerging need to find more effective therapeutic approaches for IPF patients. In Rasooli *et al*.’s study, combination therapy was performed as a new therapeutic strategy for IPF treatment (27). Accordingly, in the present study, we aimed to assess another combination strategy relying on the concomitant use of LOS and PFD. Thus, the present study aimed to assess the LOS+PFD protective effects against the destructive fibrotic effects of BLM on the A549 cell line *in vitro.*

PFD is an anti-fibrotic compound (28) and a major hurdle for BLM-induced EMT. According to research, PFD prevents EMT and fibroblast activity. Thus, it possesses great potential as a new treatment for non-small cell lung cancer (NSCLC) cell lines (29). An angiotensin II type 1 receptor antagonist, LOS, is commonly utilized to treat hypertension. It also has anti-fibrotic potential in the lung fibrosis model (13). Regarding the common target protein between LOS and PFD, the present study employed the non-toxic concentration of the LOS+PFD combination to inhibit the EMT process in A549 cells. Similar to our approach, the previous studies also used non-toxic concentrations of drugs to inhibit EMT (30, 31). 

BLM was employed as an inducer of the cellular EMT process in the A549 cell line to simulate the IPF model *in vitro. *It was shown that BLM induces oxidative stress critical to its pathogenesis in IPF. After forming a complex with O_2_ and iron, BLM generates ROS, especially superoxide and hydroxyl radicals, that bind to the DNA helix leading to its breakage and subsequent oxidative events (18). Subsequent oxidative stress can trigger the EMT process (4). Our present study shows that the BLM significantly decreased cellular SOD and CAT activity and enhanced MDA levels compared with the control group. LOS and PFD increase the activation of SOD and CAT while decreasing MDA content in A549-treated BLM. Our result confirmed the previous study presenting that BLM can disturb the normal redox state of cells by decreasing the activity of anti-oxidant enzymes and increasing lipid peroxidation (LPO) (32). Exposure to BLM increased angiotensin II in lung tissues. Angiotensin II can increase free radicals in liver fibrosis, renal injury, and myocardial infarction (33). A study reported that angiotensin II type 1 receptor blockers could postpone the damages induced by free radicals through increased SOD levels while decreasing MDA contents (13). Another study found that PFD has an anti-oxidant activity by scavenging hydroxyl and superoxide anion free radicals. Furthermore, NADPH-dependent lipid peroxidation is blocked by PFD in sheep liver in a dose-dependent manner (34). The present study used a non-toxic concentration of BLM (0.1 μg/ml) to induce EMT. Our results align with the findings of Liu *et al*. who used 0.5 μg/ml BLM to treat A549 cells without causing excessive cell death (35).

EMT is considered a central mechanism in IPF through BLM exposure, characterized by reprogramming cellular signaling pathways, loss of cellular attachments and polarity, and cytoskeleton rearrangement that leads to the manifestation of cells with the invasive phenotype (5). EMT contributes significantly to myofibroblast formation and ECM deposition in pulmonary fibrosis. EMT induction is accompanied by induction of cellular migration, up-regulating mesenchymal markers (e.g., α-SMA, Vimentin, and β-catenin), and down-regulating of epithelial markers (e.g., E-cadherin and Zo-1) in alveolar and bronchiolar epithelial cells that transform them into mesenchymal cells. Furthermore, other proteins are also up-regulated during the EMT process including zinc finger transcriptional factors such as Snail1 and Slug (36). Throughout EMT, the complex of E-cadherin and β-catenin is disrupted, and β-catenin is translocated to the nucleus (37). β-catenin, through coupling with CBP (CREB binding protein), is necessary for the transcription of α-SMA in alveolar cells (38). Vimentin is another protein involved in EMT that plays a key role in coordinating PI3K/Akt and MAPK/Erk1/2 signaling pathways (39). It was shown that β-catenin could activate Slug (40). Integrin expression is inhibited after binding Slug to promoter sequences, and cellular adhesion is reduced (41). These transcription factors (slug and snail1) also maintain the silenced state of the E-cadherin gene (42). In the current study, with the reduction in E-cadherin and Zo-1 expression and increase in α-SMA, β-catenin, Vimentin, Snail1, and Slug, A549 cells-treated BLM gradually transformed into myofibroblasts, through EMT induction. Our results are consistent with another study, as BLM-activated EMT has been found to increase the protein expression of α-SMA and Vimentin and decrease E-cadherin and ZO-1 in pulmonary fibrosis (43). It was found that LOS and PFD as a single treatment can reverse the EMT process in BLM-treated A549 cells. However, when they were used in combination, their effects were more pronounced. Similarly, Kurimoto *et al.* found that PFD could inhibit the expression of E-cadherin and increase the expression of Vimentin, thereby reversing the EMT of lung adenocarcinomas and restoring cell phenotype (44). In another study, the anti-fibrotic effects of LOS were evaluated by down-regulating the TGF-β/Smad signaling pathway and inhibition of EMT (45). 

Enhancing cellular migration is one of the important characteristics of EMT induction. Following the migration, transcription factors are activated, working as repressors of E-cadherin; thus epithelial cells become motile (46). Hence, we determined the effects of BLM treatment on cellular migration, with or without LOS+PFD. The present results demonstrated that BLM significantly increased cellular migration, and combined LOS+PFD significantly inhibited the migration of the alveolar basal epithelial cells in comparison with single therapy. It has been reported that BLM induces migration of basal alveolar epithelial cells (47). Another study reported similar results, in which cellular migration was prevented by PFD treatment after TGF-β exposure (48, 49). Also, another study showed that LOS treatment significantly decreases migration in pancreatic stellate cells (50). Regarding the common target protein between LOS and PFD, inhibiting cellular migration of combined LOS+PFD was more marked. 

C: control (medium alone); BLM: bleomycin; LOS: losartan; PFD: pirfenidone

Scale bar: 100 µm; BLM: bleomycin; LOS: losartan; PFD: pirfenidone; LOS: losartan

## Conclusion

Our findings present promising data to support the possibility of clinical studies to confirm the effects of PFD in combination with LOS to treat IPF. As far as we know, this is the first study discovering the possibilities of using PFD with the LOS *in vitro *model. Nevertheless, more studies are required to gather adequate pre-clinical information for supporting a clinical trial.

## Authors’ Contributions

A A was involved in performing and analyzing of the experiments and also writing of the manuscript. M M contributed to experimental design, the interpretation of data, and writing and revising the manuscript. MR H, S KM, and M T were involved in the experimental design and revising of the manuscript. A M was the principal author for the grant and contributed to experimental design, supervising the project, and final revision of the manuscript.

## Funding

This research was supported by Kerman University of Medical Sciences, Kerman, Iran (grant number 98000071). This study was approved by the Ethics Committee of Kerman University of Medical Sciences (IR.KMU.REC.1398.422).

## Consent for Publication

Consent for publication submission is given by all authors.

## Conflicts of Interest

No potential conflicts of interest were reported by the authors.
